# Resting State fMRI Reveals Increased Subthalamic Nucleus and Sensorimotor Cortex Connectivity in Patients with Parkinson’s Disease under Medication

**DOI:** 10.3389/fnagi.2017.00074

**Published:** 2017-04-04

**Authors:** Bo Shen, Yang Gao, Wenbin Zhang, Liyu Lu, Jun Zhu, Yang Pan, Wenya Lan, Chaoyong Xiao, Li Zhang

**Affiliations:** ^1^Department of Geriatrics, Affiliated Brain Hospital of Nanjing Medical UniversityNanjing, China; ^2^Department of Computer Science and Technology, Nanjing UniversityNanjing, China; ^3^Department of Neurosurgery, Affiliated Brain Hospital of Nanjing Medical UniversityNanjing, China; ^4^Department of Radiology, Affiliated Brain Hospital of Nanjing Medical UniversityNanjing, China

**Keywords:** Parkinson’s disease, subthalamic nucleus, sensorimotor cortex, functional connectivity, hyperdirect pathway, on-medication

## Abstract

Functional connectivity (FC) between the subthalamic nucleus (STN) and the sensorimotor cortex is increased in off-medication patients with Parkinson’s disease (PD). However, the status of FC between STN and sensorimotor cortex in on-medication PD patients remains unclear. In this study, resting state functional magnetic resonance imaging was employed on 31 patients with PD under medication and 31 healthy controls. Two-sample *t*-test was used to study the change in FC pattern of the STN, the FC strength of the bilateral STN was correlated with overall motor symptoms, while unilateral STN was correlated with offside motor symptoms. Both bilateral and right STN showed increased FC with the right sensorimotor cortex, whereas only right STN FC was correlated with left-body rigidity scores in all PD patients. An additional subgroup analysis was performed according to the ratio of mean tremor scores and mean postural instability and gait difficulty (PIGD) scores, only the PIGD subgroup showed the increased FC between right STN and sensorimotor cortex under medication. Increased FC between the STN and the sensorimotor cortex was found, which was related to motor symptom severity in on-medication PD patients. Anti-PD drugs may influence the hyperdirect pathway to alleviate motor symptoms with the more effect on the tremor subtype.

## Introduction

Parkinson’s disease (PD) is the second most common progressive neurological degenerative disorder caused by dopamine deficits in the substantia nigra pars compacta (Lees et al., 2009). Impairment of the respective functions of parallel cortico-basal ganglia-thalamo-cortical circuits causes various symptoms (DeLong, 1990; Helmich et al., 2010). The subthalamic nucleus (STN) is one of the preferred targets in deep brain stimulation (DBS) treatment of PD patients, with greater clinical benefits in motor symptom improvement than those obtained by stimulating other sites (Volkmann et al., 2004; Odekerken et al., 2013). However, the exact mechanism of this stimulation remains unknown. Therefore, STN may play a role in the motor control in PD patients (Chiken and Nambu, 2014).

In a healthy brain, the STN stimulates the internal segment of the globus pallidus, leading to increased inhibition of the ventrolateral thalamus. Consequently, the motor activity is increased within the primary somatosensory cortex (S1), primary motor cortex (M1), and premotor cortical area (Weintraub and Zaghloul, 2013). This phenomenon is an indirect pathway that is depressed by dopamine. The indirect pathway is overactive in PD patients, leading to hyperactivity of the STN (Alexander and Crutcher, 1990). Furthermore, the fast hyperdirect feedback loop from supplementary motor area and M1 cortical projections to the STN via glutamatergic neurons needs further investigation (Tewari et al., 2016).

Resting state functional MRI (rs-fMRI) is a relatively novel technique which is easily carried out in large populations. However, the biological origin and relevance of these slow neuronal activity components are still poorly understood, the latter observation that spontaneous BOLD activity is specifically organized in the resting human brain, which has generated a new avenue of neuroimaging research (Deco et al., 2011). Subsequently, rs-fMRI is also a well-accepted tool in the non-invasive study of neurological and psychiatric disorders at a network level *in vivo* (Zhang and Raichle, 2010). In off-medication PD patients, an increased functional connectivity (FC) between the STN and hand M1S1 areas was found in the non-tremor subgroup with the FC strength correlating with rigor scores (Baudrexel et al., 2011), while increased FC between these two areas was also discovered in early drug-naïve PD patients (Kurani et al., 2015). Primary data in the α- and β-frequency EEG bands showed a burst oscillatory local field activity in the STN and an increased FC between STN and motor cortical in PD patients (Hammond et al., 2007; Lalo et al., 2008). Therefore, increased oscillations in the STN may be a factual reason for the abnormal activity of the M1S1 cortex. A consistent conclusion was the increased FC of STN at different stages in off-medication PD patients.

Only two articles reported the FC of STN and motor area in normal PD patients while in the on-medication. Fernández-Seara et al. (2015) showed an increased FC between the STN and the motor cortex just like in off-medication PD patients using arterial spinlabeled (ASL) perfusion fMRI, whereas Mathys et al. (2016) did not find a change in the FC between the two areas. Aside from the different methods in these two articles, we speculate that choosing patients from a broad severity range may benefit FC change analysis, as previous research shows that a broad range of severity is needed when combining the *de novo* and moderate PD groups into the correlation analysis (Kurani et al., 2015), while Fernández-Seara et al. (2015) preferred early-state PD patients (mean HY = 1.83) and Mathys selected patients with a mean duration of 6 years. Litvak et al. (2011) found that dopaminergic medication modulated the resting beta network by combining magnetoencephalographic and subthalamic local field potential recordings. However, the correlation between decreased FC strength and decreased motor symptoms in on-medication PD patients using fMRI technology was still unknown.

Hence, in this work, we selected PD patients with different severities (HY from 1 to 4 on-medication based, duration from 1 to 18 years) to assess the change in FC of STN. We tested whether changes in FC between STN and whole brain may exist, as well as the correlation with the motor symptom, because motor symptoms exist after drug administration.

## Materials and Methods

### Participants

We conducted a prospective case – control study of 36 PD patients and 31 healthy controls in the Department of Geriatrics, Nanjing Brain Hospital between July 2015 and March 2016. Patients were included in the study if they were aged 18 years or older, satisfied the standard UK Brain Bank criteria for PD (Hughes et al., 1992), and experienced at least one of the following symptoms: severe response fluctuations, dyskinesias, painful dystonias, or bradykinesia. Exclusion criteria included history of other neurological or psychiatric diseases, and cognitive impairment based on the PDD criterion in 2007 (Dubois et al., 2007). We defined anti-Parkinsonian medication to include any drug designed to alter symptoms of PD or any drug that slows the progression of PD, levodopa equivalent daily dose (LEDD) was calculated with previous research (Tomlinson et al., 2010). All PD patients were scanned twice in off-medication and in 60–90 min after taking anti-Parkinsonian medication. Only the on-medication measurements were analyzed. All participants had written informed consent and the study was approved by the Medical Research Ethical Committee of Nanjing Brain Hospital, Nanjing, China.

### Assessment of PD Motor and Cognition Symptoms

Motor impairment in patients with PD was assessed by items of Part III (motor part) of the Unified Parkinson’s disease Rating Scale (UPDRS) and H&Y staging scale for both the “on” and “off” states. Unilateral limb tremor scores are the sum of hand tremor scores and lower limb scores from UPDRSIII. The mean tremor score was derived from the sum of items 16 and 20–26 on the UPDRS, while a mean score was derived from five postural instability and gait difficulty (PIGD) items (Stebbins et al., 2013). Patients were classified as having tremor-domain Parkinson’s disease (TD-PD) when the ratio of the mean tremor score to the mean PIGD score was ≥ 1.5 and as having PIGD-PD when this ratio was ≤ 1, others were included as having mixed subtype PD. Overall cognition condition was assessed using the Mini-Mental State Examination (MMSE) and Montreal Cognitive Assessment (MoCA) (Chen et al., 2016).

### MRI Data Acquisition Protocol

MR-imaging was carried out on a 3.0-T MR scanner system (Siemens, Verio, Germany) with an 8-channel phased-array head coil for signal reception and a whole body coil for radio frequency transmission. Subjects were instructed to lie still, relax, and not think of anything in particular, while being required to keep their eyes open to avoid falling asleep. No subject reported to have fallen asleep when routinely asked immediately after examination.

### Image Acquisition

Functional scans of the brain were acquired using a gradient echo EPI sequence with the following parameters: repetition time (TR) = 2000 ms, echo time (TE) = 25 ms, matrix size = 64 × 64, field of view (FoV) = 240 mm × 240 mm, 33 slices with 4 mm slice thickness and 0 mm inter-slice gap, and scan duration of 8 min and 6 s. Axial anatomical images were acquired using a 3D-MPRAGE sequence (TR = 1900 ms; TE = 2.48 ms; flip angle [FA] = 9°; matrix = 256 × 256; FoV = 250 mm × 250 mm; slice thickness = 1 mm; and gap = 0 mm; slices covered the whole brain, with registration and functional localization). Patients were scanned during on-state medication, resulting in a 4D data set consisting of 240 volumes of functional data for subsequent FC analysis.

### Data Preprocessing

Preprocessing was carried out using Data Processing Assistant for Resting-State fMRI (DPARSF; Chao-Gan and Yu-Feng, 2010^1^) which is based on Statistical Parametric Mapping (SPM8)^2^.

The first 10 volumes of the BOLD data for each subject were discarded, while the remaining images were corrected by realignment, accounting for head motion. Three patients with head motions exceeding 3 mm of translation, or a rotation of 3°, throughout the course of the scan were excluded from the study. The remaining functional images were coregistered to the individual T1-weighted images and were then segmented into gray matter (GM), white matter (WM), and cerebrospinal fluid (CSF) tissue maps using a unified segmentation algorithm followed by non-linear normalization into the Montreal Neurological Institute space. Resultant functional images were re-sampled to 3-mm isotropic voxels, and spatially smoothed with a Gaussian kernel full width at half maximum = 4 mm × 4 mm × 4 mm. The resulting fMRI data were band-pass filtered (0.01 < *f* < 0.08 Hz) to reduce low-frequency drift and high-frequency physiological, respiratory, and cardiac noise. Any linear trend was then removed. Subsequently, six head motion parameters and the mean time series of global activities, WM, and CSF signals were introduced as covariates into a random effects model to remove possible effects of head motion, global activities, WM, and CSF signals on the results. For each patient, the global signal is obtained by averaging the time series of voxels in all brain tissues, everyone has an unique global signal, global signals was thought as including the breath, heart rate, and other noise. So in the data preprocessing step, global signals are regressed out at the single-subject level.

### Seed Region and FC Analysis

**FIGURE 1:**
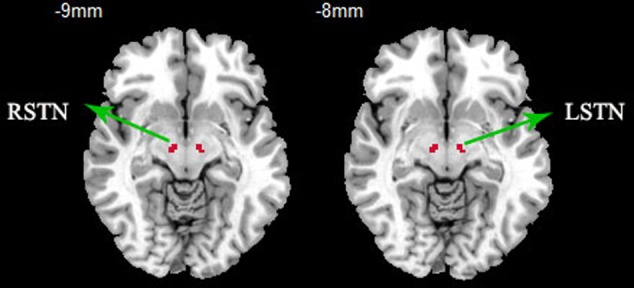
**ROI presentation of subthalamic nucleus (STN).** ROI definition of the STN, bilateral STNs were defined as regions of interest from the WFU_pickatla, the centers of the left and right STN were [-9, -12, -8] and [9, -14, -8].

The bilateral STNs were defined as regions of interest from the WFU_pickatla (Talairach brain atlas theory), which automatically generated segmented atlas region of interest templates in the MNI space (Sakurai et al., 2017). Each STN was about 81 mm^3^, the centers of the left and right STN were [-9, -12, -8] and [9, -14, -8], the location and extent of STN are displayed in **Figure 1**. The mean time series of bilateral STN were extracted. Furthermore, a voxel-wise FC analysis was performed by computing the temporal correlation between the mean time series of the combined left and right STN and the time series of each voxel within the whole brain. The correlation coefficients of each voxel were normalized to *z*-scores with Fisher’s r-to-z transformation. Therefore, a *z*-score map for the entire brain was created for the STN of each subject. Finally, the region where the significant difference between PD and HC, was used as the mask to extract the mean *Z* value for each PD patient. The correlation of *Z*-values and motor symptoms was measured with SPSS20, and motor symptoms included UPDRSIII, TD scores, PIGD scores, duration, bilateral tremor and rigidly scores, as the tremor and rigidly are the common symptoms of PD. The TD score was derived from the sum of items 16 and 20–26 on the UPDRS, while PIGD score was derived from five PIGD items.

### Statistical Analysis

One-sample *t*-tests were conducted on the *z*-score maps of the two groups. Then, between-group two-sample *t*-tests were performed within the whole brain mask, with age, gender, education, and GM volumes as covariates, to detect significant differences between the two groups, GM volumes were calculated by the SPM in the latter voxel based morphometry step. With in-group multiple comparisons, all T maps had a threshold of *p* < 0.005, while the cluster extent was calculated according to alphasim correction based on REST software (voxel-level *p* value < 0.005; cluster size: >69 voxels; determined by a Monte-Carlo simulation resulted in a cluster-level significance threshold of *P* < 0.005). Correlation between the FC strength of the bilateral STN and overall motor symptoms have been evaluated, while unilateral STN FC was correlated with contralateral motor symptoms, and motor symptoms included UPDRSIII, TD scores, PIGD scores, duration, bilateral tremor and rigidly scores, as the tremor and rigidly are the common symptoms of PD. All correlation analyses were performed using the SPSS20.0 software package.

### Voxel Based Morphometry (VBM)

To test whether the change in FC pattern was associated with the structural atrophy, gray and white matter volumes were measured based on the SPM8. VBM procedure involved the segmentation of the original structural MRI images in native space into GM, WM, and CSF tissues, then GM and WM images were normalized to templates in stereotactic space to acquire optimized normalization parameters, which were applied to the raw images. GM images were smoothed using an 8-mm full width at half maximum isotropic Gaussian kernel. The last, we employed a general linear model, using age and sex as covariates. Comparison between PD patients and healthy controls was again carried out with the two-sample *t*-test option provided in the SPSS software, with significant difference set at *p* < 0.05.

## Results

### Clinical and Neuropsychological Evaluations

A total of 31 PD patients were included in our study (excluding three patients whose head motions exceeded 3 mm of translation and another two patients who could not bear the noise of the MRI). The 31 PD patients contained varying motor symptom severity and durations, with 18 of the 31 patients being more affected at the left side of the body in terms of UPDRS III. For subsequent analyses, due to the smaller size of TD patients, patients with tremor-dominant (*n* = 5) and mixed type (*n* = 9) were pooled and referred to as the tremor subgroup (*n* = 14) similar to the previous study (Baudrexel et al., 2011). No significant differences in age, sex, education, MMSE, and MOCA were found for the three groups. **Table 1** summarizes the detailed demographic and clinical characteristics of the three groups (patients with PD and healthy controls).

**Table 1:** Demographic and neuropsychological characteristics of all subjects (on-medication).

Groups	PD (*n* = 31)	TD (*n* = 14)	PIGD (*n* = 17)	HC (*n* = 31)	*P*-value
Sex (male/female)	16/15	8/6	8/9	16/15	0.958^a^
Age, years	60.29 @ 9.03	61.07 @ 9.18	59.64 @ 9.14	59.71 @ 4.79	0.943
Education, years	11.71 @ 3.53	11.79 @ 3.85	11.64 @ 3.71	12.00 @ 2.42	0.642
MOCA	26.32 @ 2.65	26.00 @ 2.66	26.59 @ 2.69	27.03 @ 1.33	0.146
MMSE	27.71 @ 1.53	27.79 @ 1.63	27.65 @ 1.50	28.03 @ 1.49	0.405
Duration, years	6.64 @ 4.20	5.79 @ 2.33	7.35 @ 5.24	NA	0.588
H&Y stage	2.55 @ 0.97	2.43 @ 0.92	2.65 @ 1.03	NA	0.825
UPDRS	36.68 @ 14.45	32.43 @ 14.34	40.18 @ 14.00	NA	0.331
UPDRSIII	18.58 @ 9.03	16.86 @ 8.97	20.00 @ 9.11	NA	0.631
Termor scores	3.55 @ 3.64	4.86 @ 3.72	2.47 @ 2.70	NA	0.140
PIGD scores	3.84 @ 2.21	2.79 @ 1.81	4.71 @ 2.17	NA	0.050
Left lumbar tremor scores	0.81 @ 1.47	1.14 @ 1.40	0.53 @ 1.50	NA	0.514
Right lumber tremor scores	0.74 @ 1.18	1.21 @ 1.53	0.35 @ 0.61	NA	0.126
Left lumbar rigidly scores	1.84 @ 1.66	1.791.81	1.881.58	NA	0.987
Right lumbar rigidly scores	1.45 @ 1.35	1.43 @ 1.50	1.47 @ 1.28	NA	0.996
LEDD, mg/day	614.38 @ 269.62	619.46 @ 242.04	610.21 @ 297.75	NA	0.996

*MMSE, Mini-Mental State Examination; TD, tremor-dominant; PIGD, postural instability and gait difficulty; UPDRS, unified Parkinson’s disease rating scale; LEDD, levodopa equivalent daily dose; *p*^a^-value for the gender difference was obtained by chi-square test, others were obtained using one-way analyses of variance.*

### STN FC in Healthy Controls

**FIGURE 2:**
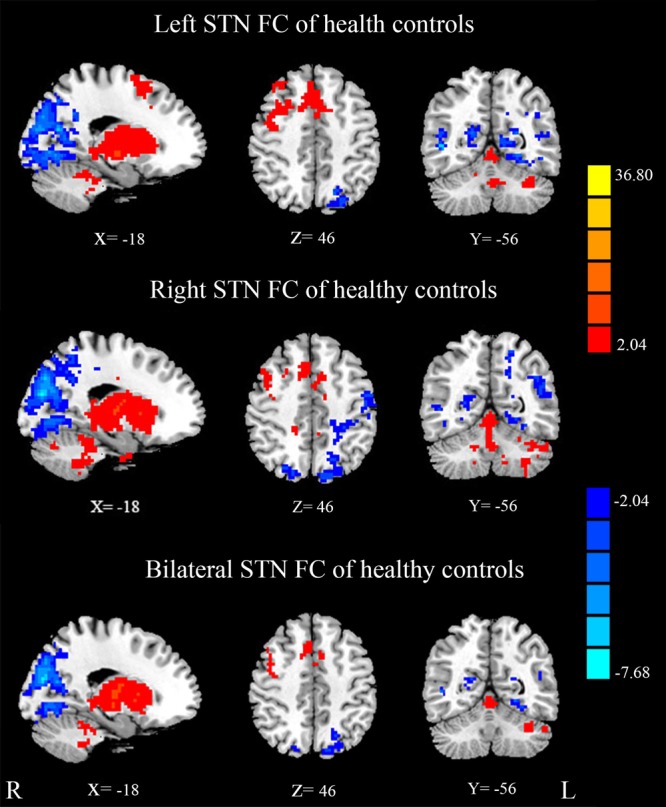
**Mean resting state functional connectivity (FC) *z*-score maps of the STN in healthy controls.** STN FC map of healthy controls, results are in MNI space, red color represents the positive correlation while the blue color represents the negative correlation.

Mean resting state FC *z*-score maps of STN in healthy controls are displayed in **Figure 2**. The correlation of left and right STN with whole brain was measured with DPARSF and REST software, as a result, the left and right STN FC pattern were compared with the two-sample *t*-test to seek the differences, and there is no difference. The result showed that most of the positive *z*-score values were found bilaterally in the brainstem, caudate nucleus, putamen, thalamus, and the cerebellum. Moreover, relatively small positive *z*-score values were found in the Frontal Lobe, which included middle frontal gyrus, superior frontal gyrus, frontal eyes field, pre-motor, and supplementary motor cortex in the right cerebrum. In contrast, negative *z*-score values were found in bilateral precuneus, which is the core area of the DMN, cuneus, lingual gyrus, middle occipital gyrus, left superior occipital gyrus, right middle temporal gyrus, left primary visual cortex, and calcarine.

### Between-Group Differences of STN FC

**FIGURE 3:**
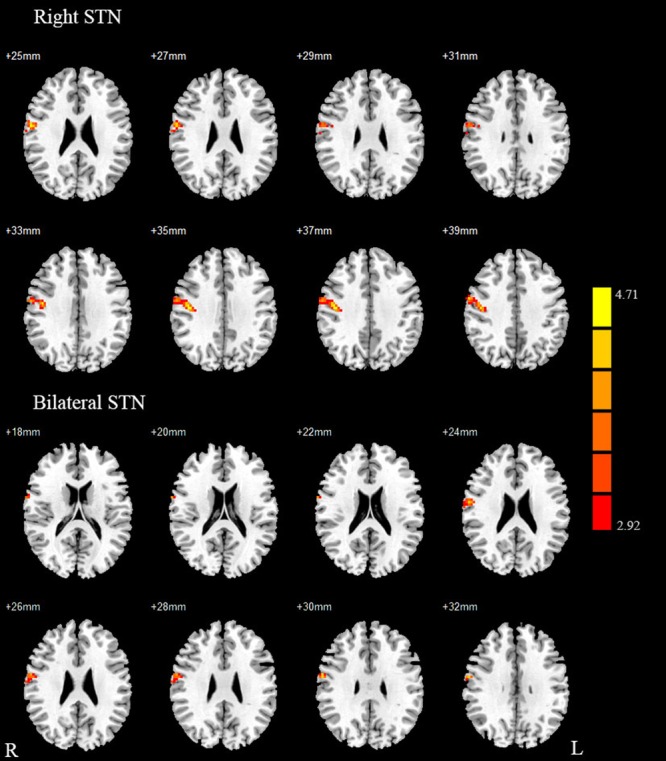
**Between Parkinson’s disease (PD) group and HC of difference in the STN resting state FC.** Between-group differences in the STN – sensorimotor cortex FC, results are in MNI space, red color represents the increased correlation while the blue color represents the decreased correlation.

**FIGURE 4:**
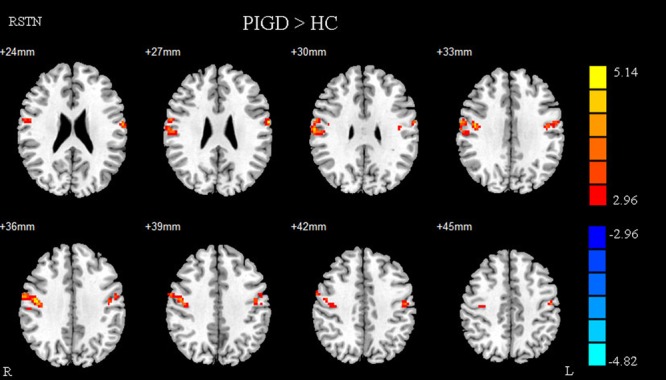
**Between postural instability and gait difficulty (PIGD) subgroup and HC of difference in the right STN resting state FC.** Between PIGD subgroup and HC of difference in the STN resting state FC, results are in MNI space, red color represents the increased correlation while the blue color represents the decreased correlation.

**FIGURE 5:**
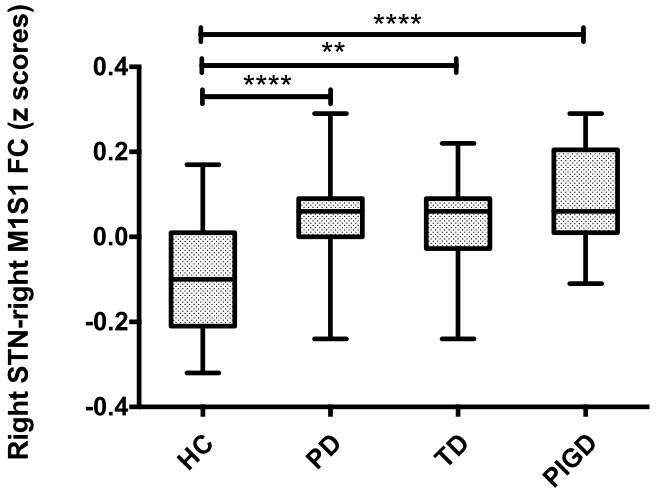
**Difference of right STN–right M1S1 FC values in PD subgroups and healthy controls.** Box plots of right STN–right M1S1 FC values in PD subgroups and healthy controls. Significant differences between healthy controls and the respective PD subgroups are indicated with (^∗^), ^∗∗^*p* = 0.002, ^∗∗∗∗^*p* < 0.0001.

Compared with healthy controls, the PD patients exhibited increased right STN FC with the right M1S1, which contains the precentral and postcentral gyrus (*p* < 0.005, cluster size: >69 voxels, multiple-comparison correction using AlphaSim in REST), while no decreased areas were found as shown in **Figure 3** and **Table 2**. Increased FC patterns of bilateral STN with the right M1S1 were also found as shown in **Figure 3** and **Table 2**. The left STN FC pattern had no change in area, while all T maps showed no decrease in area. The PIGD showed the increased FC between the right STN and bilateral M1S1 compared with the HC with on changed pattern in TD subgroup as shown in **Figure 4** and **Table 3**. *Z*-values of right STN and right M1S1 in the different groups as shown in **Figure 5**.

**Table 2:** REST group comparison results indicating increased STN FC in PD patients as compared to healthy controls.

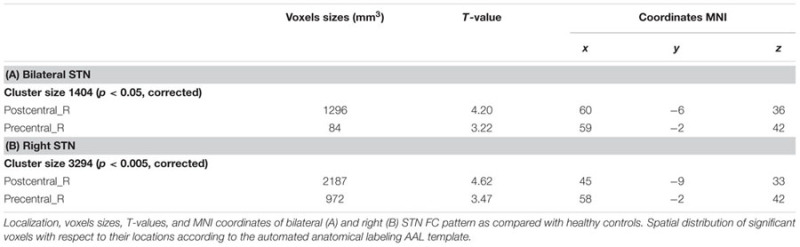

**Table 3:** REST group comparison results of right STN in PIGD patients.

	Voxels sizes (mm^3^)	*T*-value	Coordinates MNI		
**Cluster 1 (Sizes 3699, *p* < 0.005, corrected)**					
Postcentral_R	2097	5.14	45	-11	36
Precentral_R	999	3.15	47	-7	37
**Cluster 2 (Sizes 1809, *p* < 0.01, corrected)**					
Postcentral_L	1674	5.04	-62	-5	28
Precentral_L	54	3.57	-54	-5	32

*Localization, voxels sizes, *T*-values, and MNI coordinates of right STN FC pattern as compared with healthy controls. Spatial distribution of significant voxels with respect to their locations according to the AAL template. PIGD, postural instability gait difficulty.*

### STN–M1S1 FC and Motor Symptom Correlation Analysis

**FIGURE 6:**
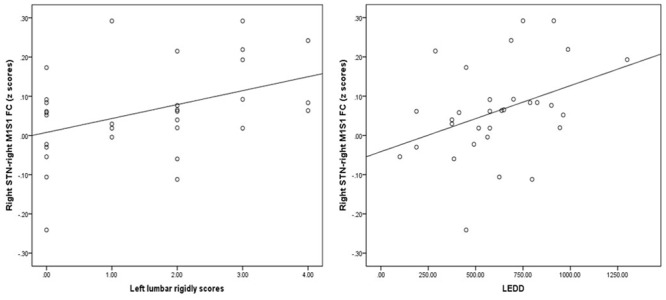
**Results of the correlation of FC values with levodopa equivalent daily dose (LEDD) and motor symptom.** Left graph showed the correlation diagram of right STN–M1S1 area FC values with corresponding UPDRS III right lumbar rigor scores (hand scores plus leg scores) in patients, right graph showed the correlation diagram of same FC values with LEDD.

Finally, the region where the significant M1S1 clustered, as a result of the between-group analysis, was used as the mask to extract the mean *Z* value for each PD patient. The *Z*-values of right STN showed no correlation with tremor and PIGD scores. By contrast, the *Z*-values of right STN and right M1S1 showed a correlation with left lumbar rigidity scores from UPDRS (*r* = 0.414, *p* = 0.021) and LEDD (r = 0.435, *p* = 0.014) in 31 PD patients (**Figure 6**). What’s more, there were no correlation of *Z*-values and motor symptoms in the PD subgroup.

### ROI Analysis of Right STN–Right M1S1 FC

A special ROI centered at the *t* maximum from the previous experiments was used as functional representation of right M1S1 for further research. This special ROI was defined by the changed area of STN FC pattern between PD and HC. It was in the right hemisphere and it’s peak coordinate was (45, –11, 36), the detailed information of M1S1 were seen in **Table 2**. Compared with the HC, both the PD subgroup showed the increased FC (TD: *p* = 0.002, PIGD: *p* < 0.001), A direct statistical comparison of the two PD subgroups under medication again yielded no significant results similar to the previous study (Baudrexel et al., 2011) (**Figure 5**).

### VBM

Voxel Based Morphometry did not reveal significant differences between patients and healthy controls for gray matter volume and WMV, with detailed information in **Table 4**.

**Table 4:** Results of VBM analysis between PD, PD subgroup, and HC groups.

	PD	TD	PIGD	HC	*F*-value	*P*
GMV (cm^3^)	543.50 ± 61.45	536.50 ± 60.40	549.26 ± 63.54	573.77 ± 59.25	1.811	0.151
WMV (cm^3^)	585.02 ± 65.62	593.80 ± 71.82	577.80 ± 61.30	577.92 ± 72.02	0.217	0.884

*GMV, Gray matter volume; WMV, white matter volume; volumes are represented as the mean ± standard deviation. For comparisons of demographics, *P*-values were obtained using one-way analyses of variance. TD, tremor-dominant; PIGD, postural instability gait difficulty.*

## Discussion

The STN is the preferred target in DBS surgery to normalize aberrant patterns of STN and to improve cardinal motor symptoms. However, the mechanism is still unclear. The current study found that the combined bilateral STN and right STN showed increased FC with M1S1 in PD patients under medication. These findings provide new evidence of increased FC between STN–M1S1. Furthermore, severe motor symptoms related to the changes in STN–M1S1 FC may also exist in PD patients despite the effects of medication.

Resting state fMRI provided a new way of viewing the hyperdirect pathway, although its mechanism remains unknown. Moreover, the overactivity of the hyperdirect pathway was proven in the off-medication PD patients and animal PD models (Dejean et al., 2008). Kahan et al. (2014) found a decrease in the effective FC of the hyperdirect pathway with STN stimulation, with the relationship between decreased hyperdirect coupling strength and improved clinical severity being particularly interesting. Local field potential recordings from the STN of patients undergoing surgery for DBS revealed strong oscillatory activity, particularly in the beta band (13–35 Hz) (Kuhn et al., 2004). STN beta oscillations were reduced by the application of levodopa and DBS (Priori et al., 2004; Kuhn et al., 2008). Furthermore, STN beta power reduction correlated with clinical improvement (Kuhn et al., 2008). The STN has also been involved in reactive global inhibition through the hyperdirect path (Vink et al., 2005; Zandbelt et al., 2013), with the increased 2.5–5 Hz phase activity of STN leading to increased response thresholds and slower responses (Tewari et al., 2016). Previous studies showed increased FC of STN with M1S1 in off- and on-medication patients. Adding our conclusions, the overactivity of the so-called hyperdirect loop and the occurrence of motor symptoms could be simultaneously depressed with the effects of medication.

Compared with the ASL research, the same conclusions regarding the increased correlation of bilateral STN and M1S1 and the similar correlation between FC and LEDD were seen, which may show the modulation of medication on brain activity (Litvak et al., 2011). Multi-model research correlating ASL, fMRI, and EEG research outputs would help us know more about the mechanism of this disease. The difference in the FC pattern was that changed areas in our research were all in right hemisphere, whereas they found the left part to be mainly altered. This may be because our patients were more onset in the left side of body since the change in FC was only based on the seed of the right STN, while no significant difference was found in the seed of the left STN. Furthermore, previous research performed right-sided surgery faster but with higher errors (Obeso et al., 2013), which means that the right STN is more likely to be involved in action inhibition (Tewari et al., 2016). This abnormal FC in the unilateral cerebral hemisphere is consistent with previous studies wherein the unilateral hemispheric basal ganglia-thalamo-cortical circuit modulated the contralateral movement (Kahan et al., 2014).

We selected unilateral STN as the seed to find its relationship to the offside motor symptom because of the unsymmetrical severity of motor symptoms. Our findings were similar to that of Baudrexel’s research wherein FC strength with right STN and right M1S1 were correlated with left-leg rigor scores. Previous studies showed an association between rigor strength and increased oscillatory activity within the STN (Kuhn et al., 2009). We found that the relationship between FC strength and rigidity scores may be because of the stability and objectivity of the rigidity symptoms, whereas tremors were always affected by mood. A little difference between our research with previous off-medication study is that the PIGD showed the most significant enhanced FC pattern. In our study, some people presented serious tremors in the off-state, which disappeared after taking anti-PD drugs. This finding is consistent with previous studies showing that tremors are more affected by drugs (Connolly and Lang, 2014), along with increased FC strength. Katz et al. (2015) found TD patients had greater mean overall motor improvement than PIGD patients after STN DBS, measured by UPDRS-III. Anti-PD drugs or DBS may have a more effect on the hyperdirect pathway of TD patients to resolve the abnormal activity of STN. Therefore, in studies involving on-state PD patients, the burst FC pattern is also related with the motor symptoms and PD subgroup showed the different hyperdirect pathway under medication.

## Conclusion

We are the first to prove the increased FC patterns and the correlations between rigidity symptoms of varying severity in PD patients under medication. Our findings further suggest that PIGD and tremor symptoms might be linked to an different coupling of these areas in on-medication PD patients. Moreover, anti-PD drugs may changed the hyperdirect pathway, thereby altering the motor symptoms.

## Author Contributions

LZ designed the study and revised it critically for important intellectual content. BS performed the research and drafted the manuscript, YG and YP helped in data analyses, WZ, CX, LL, and JZ help in clinical data collection and analyses, and made patient follow-ups, and WL edited the paper.

## Conflict of Interest Statement

The authors declare that the research was conducted in the absence of any commercial or financial relationships that could be construed as a potential conflict of interest.

## Footnotes

1 http://rfmri.org/DPARSF

2 http://www.fil.ion.ucl.ac.uk/spm
